# Seroprevalence of human respiratory syncytial virus and human metapneumovirus in healthy population analyzed by recombinant fusion protein-based enzyme linked immunosorbent assay

**DOI:** 10.1186/1743-422X-9-130

**Published:** 2012-07-02

**Authors:** Patricia Sastre, Tamara Ruiz, Oliver Schildgen, Verena Schildgen, Carmen Vela, Paloma Rueda

**Affiliations:** 1 Inmunología y Genética Aplicada S.A. (INGENASA), Madrid, Spain; 2 Institute for Pathology, Kliniken der Stadt Köln gGmbH, Private University of Witten/Herdecke, Cologne, Germany

**Keywords:** Human respiratory syncytial virus, Human metapneumovirus, Seroprevalence and immunoassay

## Abstract

**Background:**

Human respiratory syncytial virus (hRSV) and human metapneumovirus (hMPV) are two of the most frequent respiratory pathogens that circulate worldwide. Infection with either virus can lead to hospitalization of young children, immunocompromised people and the elderly.

A better understanding of the epidemiological aspects, such as prevalence of these viruses in the population will be of significant importance to the scientific community. The aim of this study was to gain some detailed knowledge on the humoral immune response to both viruses in different populations of individuals.

**Findings:**

The fusion protein (F) of hRSV and hMPV was expressed in the baculovirus and *Escherichia coli* systems, respectively, and used as antigen in two independent enzyme-linked immunosorbent assays (ELISAs) for detection of specific antibodies in human sera. The seroprevalence of each virus in a large cohort of individuals with ages ranging from 0 to 89 years old was determined. Although the general distribution of the antibody response to each virus in the different age group was similar, the prevalence of hRSV appeared to be higher than that of hMPV in most of them. The group of children with ages between 0 and 2 showed the highest seronegative rates. After this age, an increase in the antibody response was observed, most likely as the result of new infections or even due to reinfections.

**Conclusions:**

The use of these specific F-ELISAs in seroepidemiological studies might be helpful for a better understanding of the human antibody response to these viruses.

## Background

Human respiratory syncytial virus (hRSV) is the leading cause of hospitalization for respiratory tract infections in children [1,2]. More recently, human metapneumovirus (hMPV), a *Pneumovirus* belonging also to *Paramyxoviridae* family, has been isolated from children hospitalized with acute respiratory infections in The Netherlands and since, the virus has been reported worldwide [3-5]. Symptoms associated with hMPV infection are very similar to those resulting from hRSV infection, ranging from common cold to severe lower respiratory tract infections, including bronchiolitis and pneumonia. No effective vaccine is currently available towards either hRSV or hMPV, and reinfections occur throughout life [6-8].

The aim of this study was to analyze and compare the specific antibody response in human serum against hRSV and hMPV in a large cohort of individuals with ages ranging from 0 to 89 years old. The fusion protein (F) of both viruses appears as the most suitable candidate for this type of analysis since it is the most immunogenic antigen of the virion and highly conserved among different strains [9-11] . For that purpose, the F protein of hRSV and hMPV was expressed in the baculovirus and *Escherichia coli* (*E. coli*) system, respectively, and used as antigen in two independent ELISAs.

## Materials and methods

### Serum specimens

Serum samples from 1811 healthy patients were randomly collected from archives of the Institute of Virology of the University Hospital Bonn.

An ethical vote from the University of Bonn Ethical Committee permitted us to use the samples retrospectively. No written informed consent was necessary as no personal data were used and as it was an epidemiological study. All procedures were carried out in accordance to the Helsinki declaration in its present form.

### Cloning and expression of the fusion protein of hRSV and hMPV

A soluble version of the F protein of hRSV, Long strain (Fs, 24-524aa) was expressed in the baculovirus system as previously described [12].

The recombinant F protein of hMPV was prepared using the *E. coli* system. The pCR4-TOPO vector containing the F protein gene of hMPV (isolate NL/1/99) was used to amplify a soluble version of the F protein gene (Fs, nt 601–1413) by PCR with primers F + (5’-**ATG**CTAAATGTTGTGCGGCAGTTT-3’) and F- (5’-**TTA**TCCTTTTTCTGCACTGTTTAG-3’). The PCR product was further cloned in the pDEST™17 expression vector (Invitrogen) that contains an N-terminal 6xHis tag. The recombinant F protein was expressed by transformation of BL21 cells. Overnight (ON) cultures of transformed bacteria were inoculated in Luria Broth medium supplemented with 1 % ampicillin. Cultures were grown to exponential phase prior to induction with arabinose 20 % for 3 hours. The F protein was partially purified from inclusion bodies by solubilization procedures using the following buffer: 50 mM Tris–HCl, 50 mM NaCl, 0.5 mM EDTA, 5 mM TCEP, 5 % glycerol and 30 % N-Lauroylsarcosine, pH 8.

### Enzyme-linked immunosorbent assay

Detection of serum antibodies against the F protein of hRSV by ELISA has been previously published [12].

For detection of antibodies against hMPV, the recombinant F protein expressed in the *E. coli* system was used as antigen to coat 96-well microtitre plates (0,2 μg/well). After ON incubation at 4 °C, the wells were blocked and a 1/500 dilution of human serum in 0.05 % Tween in Phosphate-Buffered Saline (PBS) was incubated for 1 hour at RT. Bound antibodies were detected by incubation with peroxidase-labelled anti-human IgG and subsequent addition of the substrate tetramethylbenzidine (TMB-MAX, Neogen Corporation). The cut-off value of the assay was defined by adding 2 standard deviations (SD) to the mean optical density (OD) value of 10 negative samples [13]. These samples were previously established as negative by comparison with negative controls tested by immunofluorescence and kindly provided by Dr. Catherine Manoha (University Hospital Dijon).

## Results

### Expression of the fusion protein of hRSV and hMPV

**Figure 1:**
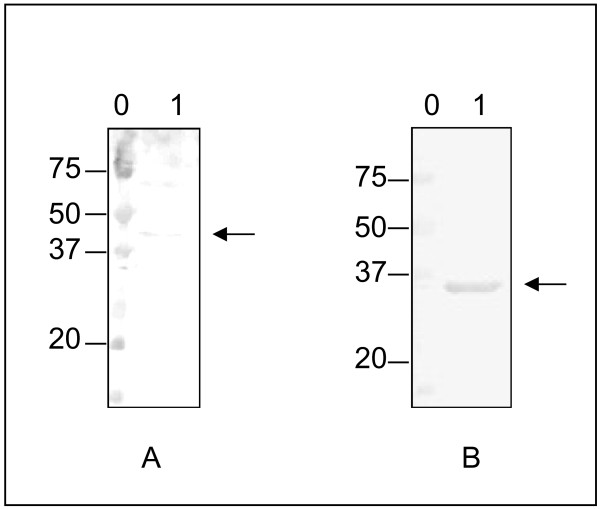
**Western blot analysis of recombinant F protein of hRSV (A) and hMPV (B).** A specific pool of positive human sera against the F protein of each virus was used to confirm the antigenicity of the recombinant proteins. Lanes 0 represent the molecular weight markers expressed in kDa and lanes 1 show the reactivity against the F protein of hRSV and hMPV, respectively. The location of the proteins is indicated with an arrow.

The F protein of hRSV and hMPV were expressed using the baculovirus and *E. coli* expression systems, respectively. A specific pool of positive human sera against the F protein of each virus was used to confirm the antigenicity of the recombinant proteins by Western blot (Figure 1A and 1B). Incubation of the nitrocellulose membranes with each pool of sera revealed a band corresponding to expected molecular mass of each recombinant protein.

### Analysis of the antibody response towards hRSV and hMPV

In order to investigate the incidence of hRSV and hMPV in the population, a set of 1811 serum samples from patients with ages ranging from 0 to 89 years of age, was included in the present study. The specific antibody response of each serum to the F protein of both viruses was measured by indirect ELISA. Each serum sample was tested at a single dilution for screening purposes. The optimal dilution was previously determined on the basis of OD yielded from the ELISA with twofold serial dilutions of control serum samples against positive and negative antigens prepared in the same conditions.

**Figure 2:**
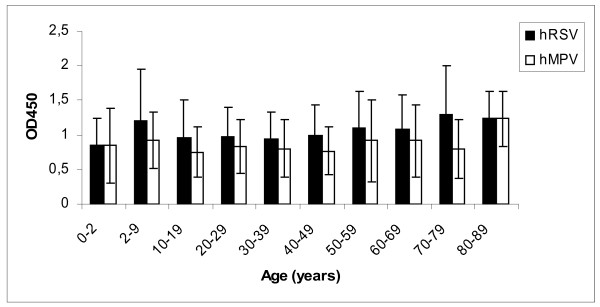
**Distribution of anti-human hRSV and hMPV antibodies in different age groups tested by F-ELISA.** Bars represent the mean optical density of the positive serum samples included in each age group plus the standard deviation.

Figure 2 shows the seropositivity against each virus in the different age groups. The high values of the SD in each age group were due to a wide range of positivity defined by the cutoff values (0.378 and 0.357 for hRSV and hMPV, respectively). Thus, samples with OD equal or higher than the cut-off, independently of the magnitude of the OD, were considered positive and sera with readings lower than the cut-off were considered negative. The percentages of seropositive and seronegative samples in each age group of patients are shown in Table 1. Although the general distribution in the different age groups for both viruses was similar, the prevalence of hRSV was higher than that of hMPV in the majority of the groups.

**Table 1:** Age Seroprevalence Profiles of hRSV and hMPV

**Age (Years)**	**Total no. samples**	**hRSV**		**hMPV**	
		**No. (%) positive**	**No. (%) negative**	**No. (%) positive**	**No. (%) negative**
0-2	80	29 (36.25)	51 (63.75)	42 (52.50)	38 (47.50)
2-9	7	5 (71.43)	2 (28.57)	6 (85.71)	1 (14.29)
10-19	43	41 (95.35)	2 (4.65)	36 (83.72)	7 (16.28)
20-29	772	733 (94.95)	39 (5.05)	707 (91.58)	65 (8.42)
30-39	417	397 (95.20)	20 (4.80)	363 (87.05)	54 (12.95)
40-49	281	269 (95.73)	12 (4.27)	244 (86.83)	37 (13.17)
50-59	134	123 (91.79)	11 (8.21)	113 (84.33)	21 (15.67)
60-69	52	50 (96.15)	2 (3.85)	45 (86.54)	7 (13.46)
70-79	19	18 (94.74)	1 (5.26)	11 (57.89)	8 (42.11)
80-89	6	5 (83.33)	1 (16.67)	4 (66.66)	2 (33.34)
Σ	1811 (100)	1670 (92.21)	141 (7.79)	1571 (86.75)	240 (13.25)

Patients with ages between 0 and 2 years old, showed the highest percentage of seronegative samples for the two viruses (63.7 % for hRSV and 47.5 % for hMPV). Then, an increase of the seropositive rates was observed in the cohort of individuals from 2 to 9 years old, with percentages of 71.4 % and 85.7 % for hRSV and hMPV, respectively. The group of patients with ages between 10 and 69 years old showed an average of seropositivity of 94.8 % for hRSV and 86.6 % for hMPV. Finally, a minor drop in the seropositivity was observed in the elderly population between 70–89 years when measuring the antibody response to hMPV. In the case of hRSV, the population between 70 to 79 years old showed similar seropositive rates to the ones of younger groups, followed by a moderate decrease in the oldest group of patients with ages between 80–89 years old.

## Discussion

Although several studies have used single recombinant viral proteins as ELISA antigens for measurement of antibody response to hRSV and hMPV [14,15], this is the first time that the same set of sera comprising a wide range of ages is tested to compare the specific antibody response against the F protein of the two viruses. To do so, the fusion protein of each virus was partially purified and used in an ELISA to detect the presence of specific antibodies in a large cohort of individuals with ages ranging from 0 to 89 years. Since the F protein of each virus was expressed in different systems and bearing in mind that post-transductional modifications do not occur in bacteria and the ones in insect cells differ from the ones in mammalian cells, the antigenicity of both proteins was first tested by Western Blot analysis with a positive pool of human sera. A band corresponding with the expected molecular size of each recombinant fusion protein was confirmed (Figure 1A and 1B).

By the age of 2 to 5 years most children have been infected at least once with both hRSV and hMPV [1,4]. In the present report, the youngest group of individuals with ages between 0 and 2 years showed the lowest antibody response to either virus. A broad range of ages are included in this group with a high percentage of children younger than 1 year old. The immaturity of the immune system of these infants could explain the low antibody level. Furthermore, the level of antibodies in this group could be even lower, since some of these sera samples may contain maternally derived antibodies, which may not represent true seropositivity due to infection. A rise in the antibody response against both viruses in the groups of individuals with ages between 2–69 years old was detected, probably as result of re-infections. Finally, the oldest group of patients from 70 to 89 years old, showed a significant antibody response towards both viruses, however they still comprise one of the groups at high risk of infection with elevated rates of morbidity [16]. Previous studies have shown that the percentage of neutralizing antibodies against hRSV in the frail people (> 80 years old) is very low, despite having a considerable level of specific antibodies against the virus. This poor neutralizing antibody response is probably one of the causes for the susceptibility of this group of individuals to develop severe disease after infection with hRSV [17]. A recent study has analyzed the neutralizing ability to inhibit hMPV replication using the same set of sera used in the present work [18]. No correlation between neutralizing ability of a given serum and antibody levels was found. Most strikingly there was an already high neutralizing capacity of the sera of the youngest group of patients although this group had the highest rate of seronegativity. For the 70–79 age group the seropositivity was 57.9 % versus 90 % neutralization. The same was true for the other age groups in which the presence of antibodies does not necessarily mean that there is also a high level of neutralizing antibodies. Although neutralizing ability is commonly associated with the presence of neutralizing antibodies in a serum sample, in the Lusebrink et al. study the authors claim that is not solely the neutralizing antibodies which inhibit hMPV replication but also other neutralizing factors, such us interferon. Taking this into consideration it is not surprising that both studies do not correlate, although the set of sera used for both studies was identical. In concert with the study by Lüsebrink et al. it appears likely that neutralizing antibodies play a minor role in the control of hMPV and hRSV infections in humans, a hypothesis that is supported by the fact that virtually all approaches to develop a potent and long lasting vaccine against both viruses have failed so far.

In the present study the F protein of NL/1/99 strain belonging to the B1 sublineage of hMPV and the F protein of the Long strain of subgroup A of hRSV have been used. Although, as mentioned in the introduction, the variability of the F protein belonging to the different subgroups and sublineages of hRSV and hMPV is low, in the developed ELISAs, some anti-F antibodies could be disregarded and therefore false negative results could be potentially obtained in both cases. However, it is well known that anti-hRSV antibodies directed against the F protein are cross-reactive for strains of both subtypes (A and B) [19], and studies on hMPV using the F protein as a subunit vaccine have indicated similar antibody reactivity patterns against homologous and heterologous hMPV infections [20,21].

Finally, as shown in Table 1, most age groups showed that the prevalence of hRSV was higher than that of hMPV, however children with ages from 0 to 9 years old had higher rates of seropositivity to hMPV than to hRSV. Several groups have studied the seroprevalence of hMPV and hRSV in young children, with different results. For instance, in The Netherlands all children have been infected with hMPV by the age of 5 [4]. In Canada, the majority of patients over 16 years old were seropositive for hMPV [5]. In Japan, Ebihara et al found that seropositive rates of hMPV were lower than those of hRSV in all children between 1 month and 5 years and a recent study in China showed that seropositive rates of hMPV in children 6 months to 6 years of age were significatively lower than seropositive rates of hRSV [22,23]. This variety of results may reflect different infection patterns between the two viruses, but also different seroprevalence patterns between geographical locations.

In summary, the present study shows that the distribution of hRSV and hMPV in the general healthy population in the area of Bonn (Germany) is very similar, with hRSV infections more frequent that those of hMPV in most age groups. This kind of analysis appears to be an appropriate instrument for seroepidemiological studies, although it should be used in combination with neutralization test for a more comprehensive analysis.

## Abbreviations

hRSV, Human respiratory syncytial virus; hMPV, Human metapneumovirus; F, Fusion protein; E. coli, Escherichia coli; ELISA, Enzyme-linked immunosorbent assay; OD, Optical density; SD, Standard deviation.

## Competing interests

The authors declare that they have no competing interests.

## Authors’ contributions

PS and PR designed the experiments and analyzed the results, TR and PS performed the experiments, VS provided the serum samples and designed experiments, PR, OS and CV contributed in critical discussion of the results, writing of the manuscript and supervised different parts of the study.
